# Age-Specific Seroprevalence of Human Parvovirus B19 in Thailand: A Cross-Sectional Study Across Four Geographic Regions with Global Comparison

**DOI:** 10.3390/tropicalmed11100291

**Published:** 2026-10-13

**Authors:** Pornjarim Nilyanimit, Jiratchaya Puenpa, Nungruthai Suntronwong, Sirapa Klinfueng, Ritthideach Yorsaeng, Thananrat Thongmee, Yong Poovorawan

**Affiliations:** 1 Department of Pediatrics, Center of Excellence in Clinical Virology, Faculty of Medicine, Chulalongkorn University, Bangkok 10330, Thailand; mim_bhni@hotmail.com (P.N.); jiratchaya.ph@chula.ac.th (J.P.); suntronwong.n@gmail.com (N.S.); sirapa.klinfueng@gmail.com (S.K.); ritthideach.yor@gmail.com (R.Y.); tata033@hotmail.com (T.T.); 2 The Royal Society of Thailand, Sanam Sueapa, Bangkok 10300, Thailand

**Keywords:** human parvovirus B19, seroprevalence, anti-B19V IgG, age-specific immunity, Thailand, seroepidemiology

## Abstract

Human parvovirus B19 (B19V) usually causes self-limiting infection but can cause severe complications in pregnancy, chronic hemolytic disease, and immunocompromise. Contemporary data on B19V immunity in Southeast Asia remain limited. We conducted an age-stratified, cross-sectional serosurvey of 430 residual specimens from four Thai provinces (April to August 2024) and compared findings against published age-specific data from 13 countries. Anti-B19V IgG was measured by quantitative ELISA, with seropositivity defined as 5.5 IU/mL or higher. Overall, 87 of 430 participants (20.2%; 95% CI, 16.5 to 24.3%) were seropositive, with a non-monotonic age pattern: 31.7% among children aged 0 to 5, falling to 4.7% at 6 to 12 years, then rising to 51.2% among those 60 and older. A serocatalytic model estimated an average constant force of infection of 8.5 per 1000 person-years beyond age 6; the present data did not provide evidence for the concentrated school-age transmission peak reported in Japan and Europe. From school age onward, Thai seroprevalence was toward the lower end of published estimates, a pattern shared with other Southeast and East Asian settings, although differences in assays, sampling frames, and study periods limit direct comparison. Seroprevalence was similarly elevated among children aged 1 to 2 and 3 to 5 years, and was therefore not concentrated in infancy; most seropositive young children had IgG concentrations close to the assay cut-off, and the explanation for this pattern remains uncertain. No significant differences by sex or province were observed. These findings suggest that a substantial proportion of adolescents and adults in the populations sampled lack detectable anti-B19V IgG and may remain susceptible to infection.

## 1. Introduction

Human parvovirus B19 (B19V), a member of the genus *Erythroparvovirus* within the family *Parvoviridae*, is a small, non-enveloped, single-stranded DNA virus with a marked tropism for erythroid progenitor cells [1]. Since its discovery in 1975 [2], B19V has been recognized as a globally distributed pathogen that causes a broad spectrum of clinical manifestations [3]. In immunocompetent children, primary infection most commonly presents as erythema infectiosum (fifth disease), whereas adults frequently experience arthropathy or acute polyarthritis [3,4]. Infection is often asymptomatic, but B19V may cause serious disease in some populations, such as transient aplastic crisis in people with an underlying hemolytic disorder [5,6], chronic anemia in immunocompromised patients [3,5], and hydrops fetalis or fetal loss after maternal infection during pregnancy [7,8].

Human parvovirus B19 is mainly transmitted through respiratory secretions, but can also be transmitted vertically from mother to fetus and by blood or blood products [3,9]. Following primary infection, lifelong IgG antibodies generally develop and confer durable immunity [3,5]. Thus, age-specific seroprevalence reflects the cumulative exposure in a population and provides valuable information about the force of infection, transmission dynamics and proportion of susceptible individuals in different age groups.

The seroepidemiology of B19V varies considerably across geographic regions and populations. Previous studies have reported overall IgG seroprevalence ranging from approximately 30% to over 80%, with substantial differences in the age at which immunity is acquired [10,11,12,13]. In many temperate countries, seropositivity increases rapidly during childhood because of transmission in schools and daycare settings [10,11], whereas in some tropical and subtropical regions, infection appears to occur later in life, resulting in a larger proportion of susceptible adolescents and women of reproductive age [14,15,16]. These regional differences have important public health implications because susceptibility among pregnant women is associated with an increased risk of adverse fetal outcomes.

Although numerous serological studies have been conducted worldwide, most were performed decades ago [17,18] or were limited to selected populations such as blood donors [19,20], pregnant women [8], or hospital-based cohorts [12,21]. Contemporary population-based data encompassing the full age spectrum remains limited, particularly in Southeast Asia. Furthermore, direct comparisons of age-specific immunity between Thailand and other countries have been scarce, making it difficult to determine whether the epidemiological pattern in Thailand resembles those reported from temperate regions or other tropical settings.

Thailand has experienced substantial demographic, socioeconomic, and public health changes over the past several decades, including declining birth rates, urbanization, improved sanitation, and changing social contact patterns, all of which may influence the transmission dynamics of childhood infectious diseases [22,23,24,25]. Updated serological data from different regions of Thailand are therefore needed to characterize the current epidemiology of B19V infection and to identify age groups with low levels of detectable immunity.

In this study, we conducted a cross-sectional seroepidemiological survey of human parvovirus B19 among apparently healthy individuals aged 6 months to 80 years from four provinces, one in each of Thailand’s four geographic regions. We determined age-specific IgG seroprevalence, compared seroprevalence among the four provinces, and compared the epidemiological profile observed in Thailand with published data from countries worldwide to place the findings in a global context.

## 2. Materials and Methods

### 2.1. Study Design and Ethical Approval

This cross-sectional seroepidemiological study was conducted using residual serum specimens from four provinces obtained from the project entitled “Evaluation of the Impact of Hepatitis B Vaccine Immunization Program as Part of the Expanded Program on Immunization (EPI) After 30 Years of Implementation and Seroprevalence of Hepatitis A, B, and C in Thailand” [25,26,27]. The parent study was approved by the Institutional Review Board of the Faculty of Medicine, Chulalongkorn University (IRB No. 787/66), and all procedures were performed in accordance with the ethical principles of the Declaration of Helsinki and relevant national guidelines. Residual anonymized serum samples remaining after completion of the original study were used exclusively for this investigation.

The use of these residual specimens for parvovirus B19 seroprevalence testing was reviewed and approved separately by the Institutional Review Board of the Faculty of Medicine, Chulalongkorn University (IRB No. 291/69), in accordance with the Declaration of Helsinki and Good Clinical Practice guidelines. The specimens were entirely anonymized residual samples with no identifiable patient information, and therefore the IRB waived the need for individual informed consent.

### 2.2. Study Population

**Figure 1:**
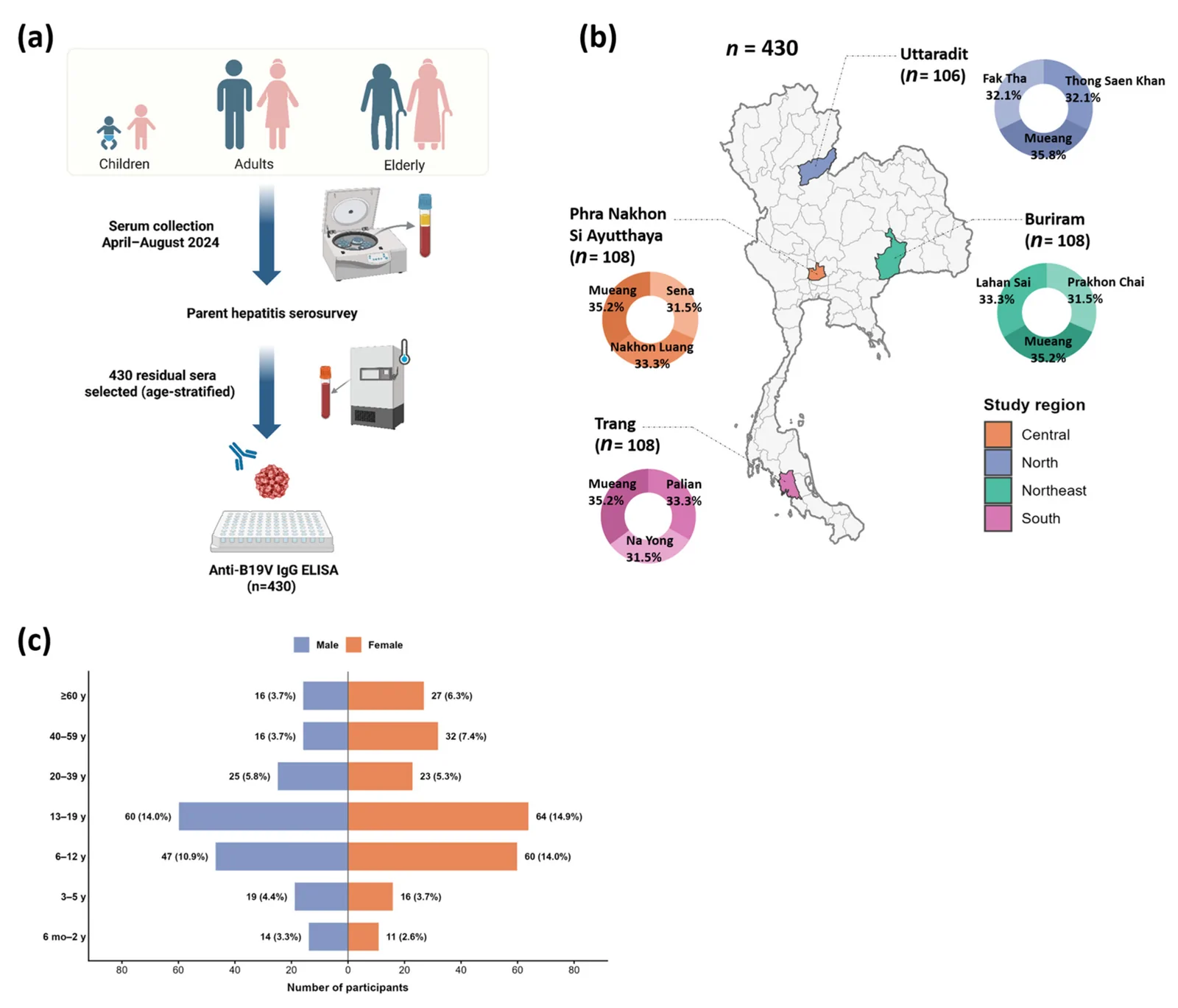
Study design, sampling framework, and demographic distribution of study participants. (**a**) Overview of specimen collection and testing workflow. (**b**) Geographic distribution of study participants across four provinces, one from each of the four regions of Thailand: Uttaradit (North, *n* = 106), Phra Nakhon Si Ayutthaya (Central, *n* = 108), Buri Ram (Northeast, *n* = 108), and Trang (South, *n* = 108). Donut charts show the proportion of participants contributed by each district within each province. (**c**) Age- and sex-specific distribution of study participants (*n* = 430), presented as a population pyramid showing the number and percentage of male and female participants in each age group.

Residual serum specimens were obtained from the parent hepatitis serosurvey conducted between April and August 2024, which included apparently healthy individuals aged 6 months to 80 years recruited using an age-stratified sampling strategy in four provinces, one from each of Thailand’s four geographic regions. Serum samples had been stored at −20 °C until analysis. Specimens for the present study were selected from all four provinces of the parent survey (*n* = 6069 participants; Figure 1a): Uttaradit Province (Northern region, *n* = 106), Phra Nakhon Si Ayutthaya Province (Central region, *n* = 108), Buriram Province (Northeastern region, *n* = 108), and Trang Province (Southern region, *n* = 108) (Figure 1b).

The sample size was calculated separately for each age group using the formula for estimating a single proportion

*n* = Z^2^P(1 − P)/d^2^

where Z = 1.96 for 95% confidence, d = 0.08 (absolute precision), and P was the age-specific anti-B19V IgG seroprevalence previously reported in Thailand [28]: 11.9% for ages 0 to 6 years, 19.1% for 7 to 12 years, 25.0% for 13 to 19 years, and 30.3% for 20 years and older. This yielded minimum sample sizes of 63, 93, 113, and 127, respectively (total, 396), which were increased by 10% to allow for specimens with insufficient volume, giving a target of approximately 436 specimens. Specimens were selected using stratified random sampling by province and age: an allocation of approximately 108 specimens per province was set before sampling, and within each province, specimens were allocated across age groups according to the calculated age-specific sample sizes and randomly selected within each age stratum. Sex was not used as a stratification variable. A total of 430 specimens were included in the analysis.

Participants were categorized into six age groups to evaluate age-specific seroprevalence: 6 months–5 years, 6–12 years, 13–19 years, 20–39 years, 40–59 years, and ≥60 years.

### 2.3. Laboratory Analysis

Serum samples were tested for immunoglobulin G (IgG) antibodies against human parvovirus B19 using the Anti-Parvovirus B19 ELISA (IgG) kit (EUROIMMUN Medizinische Labordiagnostika AG, Luebeck, Germany; product code EI 2580-9601 G), a quantitative enzyme-linked immunosorbent assay based on microtiter wells coated with recombinant parvovirus B19 antigen. All steps from sample pipetting, reagent dispensing, incubation, and washing to photometric measurement were performed on the EUROIMMUN Analyzer I-2P (EUROIMMUN, Luebeck, Germany), a fully automated ELISA processing system (version 1.11.3) according to the manufacturer. Absorbance was read at 450 nm and IgG concentrations were automatically calculated from the calibration curve and expressed quantitatively in international units per milliliter (IU/mL). Results were interpreted according to the manufacturer’s instructions: <4 IU/mL was defined as seronegative, 4 to <5.5 IU/mL as borderline, and ≥5.5 IU/mL as seropositive. For the primary analysis, seropositivity was defined as ≥5.5 IU/mL; the 16 samples in the borderline range were classified as non-seropositive in the primary analysis, consistent with the manufacturer’s guidance that a secure evaluation is not possible in this range. Quality control for each run was performed by using the manufacturer’s positive and negative controls and calibrators, according to the standard operating procedure of the analyzer. According to the manufacturer, the assay has a sensitivity and specificity of 100% and showed no cross-reactivity with antibodies against cytomegalovirus, herpes simplex virus type 1, influenza A and B viruses, measles, mumps, respiratory syncytial virus, rubella, or parainfluenza viruses.

### 2.4. Global Data Analysis

To place the findings of this study in a global context, a non-systematic, targeted literature review was conducted using PubMed, Scopus, and Google Scholar. Studies published in English between January 1990 and September 2026 reporting age-specific IgG seroprevalence of human parvovirus B19 were identified using combinations of the search terms parvovirus B19, seroprevalence, seroepidemiology, IgG, and antibody prevalence, supplemented by manual screening of reference lists of retrieved articles. The search was last updated on 27 September 2026, during revision of the manuscript. Studies were included if they reported seroprevalence stratified across at least three age groups in a general, population-based, community-based, or blood-donor population; where no such study was identified for a country, studies based on laboratory testing of referred samples were considered. Studies restricted to pregnant women, immunocompromised patients, or outbreak investigations were excluded. When more than one eligible study was available for a given country, the study with the most complete age stratification and, secondarily, the largest sample size was selected. Thirteen studies, one per country, met these criteria and were included in the international comparison, spanning five continents. For each study, country, sampling period, sample size, age strata, assay platform, and seropositivity cutoff were recorded alongside age-specific seroprevalence estimates, extracted directly from the reported tables or text of each publication, except for one study that reported age-specific data graphically only, for which values were digitised from the published figure. Age strata in infancy reflecting maternally derived antibody were not plotted, and seroprevalence reported by single year of age was pooled into broader age bands. Because reported age bands varied across studies, estimates were plotted at the approximate midpoint age of each reported stratum rather than re-binned into common age categories. Because assay platforms, cutoff values, and sampling frames also varied across studies, comparisons between the present study and previously published data are descriptive and qualitative rather than a formal meta-analysis.

### 2.5. Statistical Analysis

Seroprevalence was calculated as the proportion of participants positive for anti-human parvovirus B19 IgG antibodies (≥5.5 IU/mL; borderline results, 4 to <5.5 IU/mL, *n* = 16, were classified as non-seropositive in the primary analysis) and presented with 95% confidence intervals (95% CIs) calculated using the Clopper-Pearson exact method. In sensitivity analyses, borderline results were either reclassified as seropositive or excluded, and overall and age-specific seroprevalence and the constant force of infection were re-estimated. Geometric mean concentrations (GMC) of IgG were calculated from log10-transformed antibody concentrations, with 95% CIs derived from the log-transformed values and back-transformed to the original scale. Differences in seroprevalence among sex and provinces within each age stratum were analyzed using the chi-square test; Fisher’s exact test (2 × 2 tables) or a Monte Carlo chi-square test with 20,000 replicates (2 × 4 tables) was used instead when any expected cell count was less than 5. Pairwise differences in IgG concentration distributions across age groups were assessed using the Kruskal–Wallis test followed by Dunn’s post hoc test with Bonferroni adjustment for multiple comparisons. Differences in seroprevalence across age groups were assessed using the chi-square test. Because seroprevalence among children aged 0 to 5 years departed from an age-monotonic pattern, the trend in seroprevalence across age groups from 6 years onward was assessed using the Cochran–Armitage test for trend, and the association between age and seropositivity was quantified by logistic regression with age as a continuous variable. These analyses were performed in R version 4.4.2 [29].

The force of infection was estimated using a serocatalytic model assuming lifelong immunity and a constant force of infection, fitted as a binomial generalised linear model with a complementary log-log link and log(age) as an offset, restricted to participants aged 6 years and older because seroprevalence declined between the 3 to 5 and 6 to 12 year groups. This restriction was a post hoc decision made after the observed age pattern was examined, and the analysis should therefore be regarded as exploratory. Piecewise-constant (age bands 0 to <6, 6 to <20, 20 to <40, and ≥40 years) and Farrington age-dependent (with γ fixed at 0) models were fitted as sensitivity analyses by maximum likelihood. All three models were fitted to the same analytic dataset of participants aged 6 years and older (*n* = 370). In each model, the force of infection was assumed to operate from birth, so that the cumulative hazard at a given age included exposure accumulated before age 6 years. Accordingly, the 0 to <6 year band of the piecewise-constant model represents the force of infection experienced before age 6 by participants in the analytic dataset; it was estimated indirectly and did not use data from children younger than 6 years. The piecewise-constant model, in which the constant model is nested (all four band-specific rates constrained to be equal), was compared with the constant model using a likelihood ratio test with 3 degrees of freedom. Because the Farrington model is not nested within the constant model, all three models were additionally compared using the Akaike and Bayesian information criteria (AIC and BIC), with differences of less than 2 units considered to indicate no meaningful difference in fit. Bootstrap confidence intervals (2000 resamples) were calculated for the piecewise estimates and for the age at peak force of infection, which is reported as the bootstrap median. These models were implemented in Python (version 3.13) using the SciPy and statsmodels packages. A two-sided *p* value of <0.05 was considered statistically significant for all analyses.

### 2.6. Generative AI and AI-Assisted Technologies in the Writing Process

Generative AI tools (GPT-4o and Claude Sonnet 4.5) were used solely for language refinement, readability enhancement, and translation from Thai into English. All intellectual content, concepts, and findings were independently conceived and developed by the authors. The authors thoroughly reviewed the final manuscript to confirm its accuracy, integrity, and compliance with academic standards.

## 3. Results

### 3.1. Parvovirus Seroprevalence in Thailand, 2024

A total of 430 participants were included in this study, with a mean age of 22.6 years (SD 20.1) (Table 1). Women outnumbered men overall (233 vs. 197), and the sex ratio was reasonably balanced within each age stratum (Table 1; Figure 1c). The 6–12 and 13–19 year groups contributed the largest number of participants (107 and 124, respectively), while the group aged 60 years and older was the smallest (*n* = 43).

**Figure 2:**
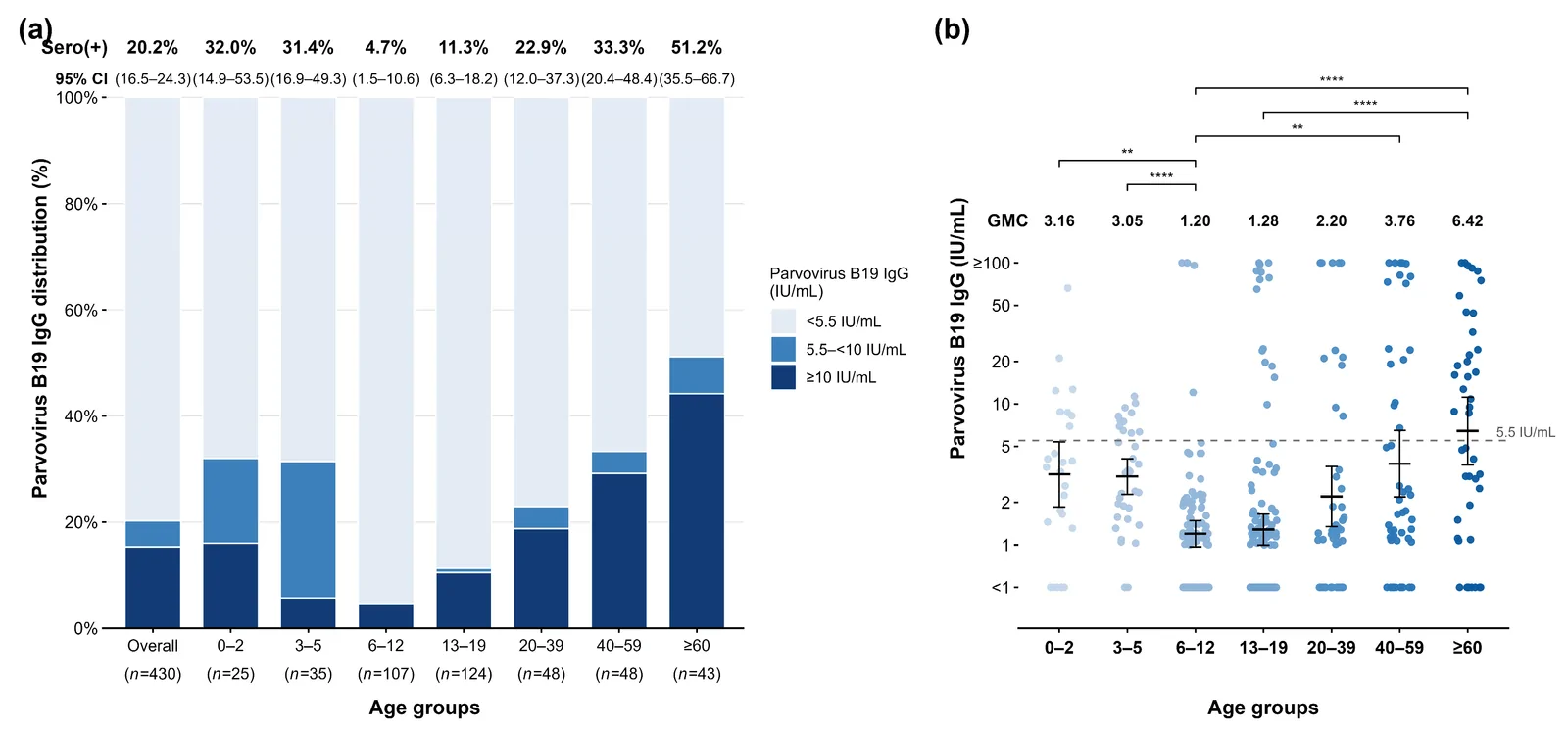
Seroprevalence and geometric mean concentrations (GMC) of anti-parvovirus B19 IgG across age groups in the general population in Thailand, April to August 2024. (**a**) Distribution of anti-parvovirus B19 IgG concentrations by age group. Seropositivity with Clopper-Pearson exact 95% confidence intervals is shown above each bar. (**b**) Individual IgG concentrations on a log10 scale. Horizontal black dashes indicate the geometric mean concentration (GMC) with 95% confidence intervals; GMC values are printed above each group. Values below the lower limit of quantification (<1 IU/mL, *n* = 145) were assigned 0.5 IU/mL, and values above the highest calibrator (>100 IU/mL, *n* = 16) were assigned 100 IU/mL; GMC in the older age groups is therefore a conservative estimate. The dashed horizontal line marks the 5.5 IU/mL seropositivity threshold. Brackets show pairwise differences in IgG concentration distributions (Kruskal–Wallis followed by Dunn’s post hoc test with Bonferroni adjustment); ** *p* < 0.01, **** *p* < 0.0001. Pre-specified comparisons are shown; the complete pairwise table is provided in Supplementary Table S1.

As shown in Figure 2a, anti-parvovirus B19 IgG was detected in 87 of the 430 serum samples tested, giving an overall seroprevalence of 20.2% (95% CI 16.5–24.3). Age-specific seroprevalence followed a non-monotonic pattern rather than a simple monotonic rise with age. Seropositivity was relatively high among young children, at 31.7% in the 0–5 year group, then fell sharply to a nadir of 4.7% in the 6–12 year group before increasing progressively across the remaining age strata: 11.3% in the 13–19 year group, 22.9% in the 20–39 year group, 33.3% in the 40–59 year group, and 51.2% in participants aged 60 years and older, the highest seroprevalence observed in the study (Figure 2a). The geometric mean antibody concentration (GMC) followed the same pattern, falling from 3.10 IU/mL in the 0–5 year group to a low of 1.20 IU/mL in the 6–12 year group, then rising steadily to a peak of 6.42 IU/mL in the group aged 60 years and older (Figure 2b). Because this population-level GMC included seronegative participants, it largely reflects the age-specific proportion seropositive. Among seropositive participants only, GMC did not increase with age from 6 years onward (Kruskal–Wallis test, *p* = 0.27). From 6 years onward, seroprevalence increased significantly with age (Cochran–Armitage test for trend, z = 7.39, *p* < 0.001), with an odds ratio for seropositivity of 1.57 (95% CI, 1.38 to 1.78) per 10-year increase in age.

### 3.2. Demographic and Geographic Determinants of Seropositivity

As summarized in Table 2, seroprevalence did not differ significantly between males and females within any of the six age strata (all *p* > 0.05), although a non-significant trend toward higher seropositivity among males was observed in the 13–19 year group (10 of 60 males vs. 4 of 64 females; *p* = 0.067). Similarly, no significant difference in seropositivity was observed among the four provinces included in this study in any age group. The largest difference between provinces, though still not statistically significant, was seen in the 40–59 year group (*p* = 0.220), in which seropositivity was highest in Trang (7 of 12) compared with Phra Nakhon Si Ayutthaya (2 of 12), Buri Ram (4 of 12), and Uttaradit (3 of 12). Given the small number of participants in each province within age strata (as few as 10 to 16), these comparisons had limited statistical power to detect differences, and the absence of statistically significant differences should not be interpreted as evidence of similar seroprevalence across provinces or between sexes. In the youngest, 0–5-year group, seropositive children were somewhat more frequent in Buri Ram (7 of 16) and Trang (3 of 15) than in Phra Nakhon Si Ayutthaya (3 of 15) and Uttaradit (6 of 14), although this difference also did not reach statistical significance (*p* = 0.294, Monte Carlo chi-square test).

### 3.3. Force of Infection and Age-Dependent Transmission Dynamics

**Figure 3:**
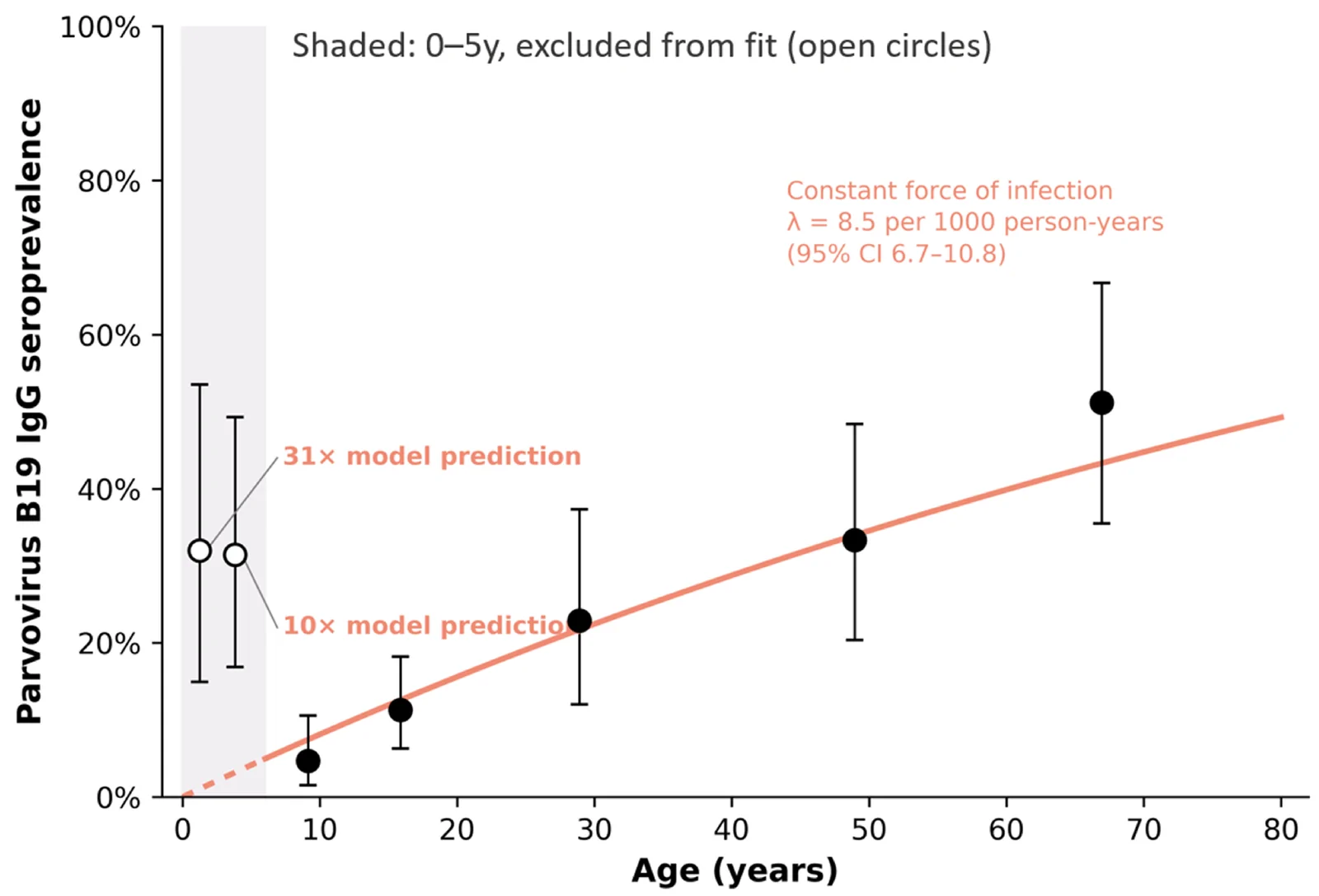
Age-specific parvovirus B19 IgG seroprevalence and fitted serocatalytic model. Filled circles show observed seroprevalence by age group, plotted at the observed mean age of the group, with Clopper-Pearson exact 95% confidence intervals. The solid coral curve is a constant force-of-infection model, 1 − exp(−λa), fitted to participants aged 6 years and older (λ = 8.5 per 1000 person-years; 95% CI, 6.7–10.8); the dashed extension shows the model-predicted seroprevalence extrapolated below that age. The shaded area indicates the age range excluded from the fit, shown with open circles, because seroprevalence declined between the 3–5 and 6–12 year groups, incompatible with the assumption of lifelong immunity. Labels give the ratio of observed to model-predicted seroprevalence in the excluded groups; these ratios reflect the discrepancy between the observed data and the model extrapolation.

Among participants aged 6 years and older (*n* = 370), a constant force of infection of 8.5 per 1000 person-years (95% CI, 6.7 to 10.8) described the age-seroprevalence profile well (Figure 3). Allowing the force of infection to vary across age bands did not improve on the constant model (likelihood ratio test, *p* = 0.335). The Farrington age-dependent model had an AIC similar to that of the constant model (303.10 vs. 303.26) and a higher BIC (310.93 vs. 307.17), indicating no meaningful improvement in fit (Supplementary Table S2). The Farrington model placed the peak force of infection at a bootstrap median age of 25.1 years, although with a wide 95% CI (14.6 to 58.2 years), and none of the 2000 bootstrap replicates placed the peak before age 10 years (Supplementary Figure S1). Thus, the present data did not provide evidence for a concentrated school-age peak in the force of infection.

Children aged 0 to 5 years were excluded from model fitting because their seroprevalence declined with age, which is incompatible with the lifelong-immunity assumption of the serocatalytic model. When the fitted model was extrapolated to these ages, observed seroprevalence was approximately 31-fold and 10-fold higher than the model-predicted values among children aged 0 to 2 and 3 to 5 years, respectively (Supplementary Table S3). These ratios reflect the discrepancy between the observed data and the model extrapolation, rather than a direct measure of higher transmission in these age groups.

When children aged 0 to 5 years were further subdivided, seroprevalence was 25.0% (1/4; 95% CI, 0.6 to 80.6) among infants younger than 1 year, 33.3% (7/21; 95% CI, 14.6 to 57.0) among children aged 1 to 2 years, and 31.4% (11/35; 95% CI, 16.9 to 49.3) among those aged 3 to 5 years (Supplementary Figure S2A; Supplementary Table S4). Seroprevalence did not differ between children aged 1 to 2 and 3 to 5 years (Fisher’s exact test, *p* = 1.00), and IgG concentration distributions were similar across the three subgroups (Kruskal–Wallis test, *p* = 0.97), indicating that seropositivity was not concentrated in infancy. Among the 19 seropositive children aged 0 to 5 years, 13 (68.4%) had IgG concentrations between 5.5 and <10 IU/mL, including 9 of 11 seropositive children aged 3 to 5 years, whereas all five seropositive children aged 6 to 12 years had concentrations of 10 IU/mL or higher (Supplementary Figure S2B). Five of the 16 borderline results (4.0 to <5.5 IU/mL) were also observed in children aged 0 to 5 years.

In sensitivity analyses, reclassifying the 16 borderline results as seropositive increased overall seroprevalence from 20.2% to 24.0% (95% CI, 20.0 to 28.3) and the constant force of infection from 8.5 to 10.1 per 1000 person-years (95% CI, 8.1 to 12.6), whereas excluding them yielded an overall seroprevalence of 21.0% (95% CI, 17.2 to 25.3) and a force of infection of 8.9 per 1000 person-years (95% CI, 7.0 to 11.3). The age pattern of seroprevalence was unchanged under both approaches (Supplementary Table S5).

### 3.4. Comparison with Previous Thai and International Serosurveys

**Figure 4:**
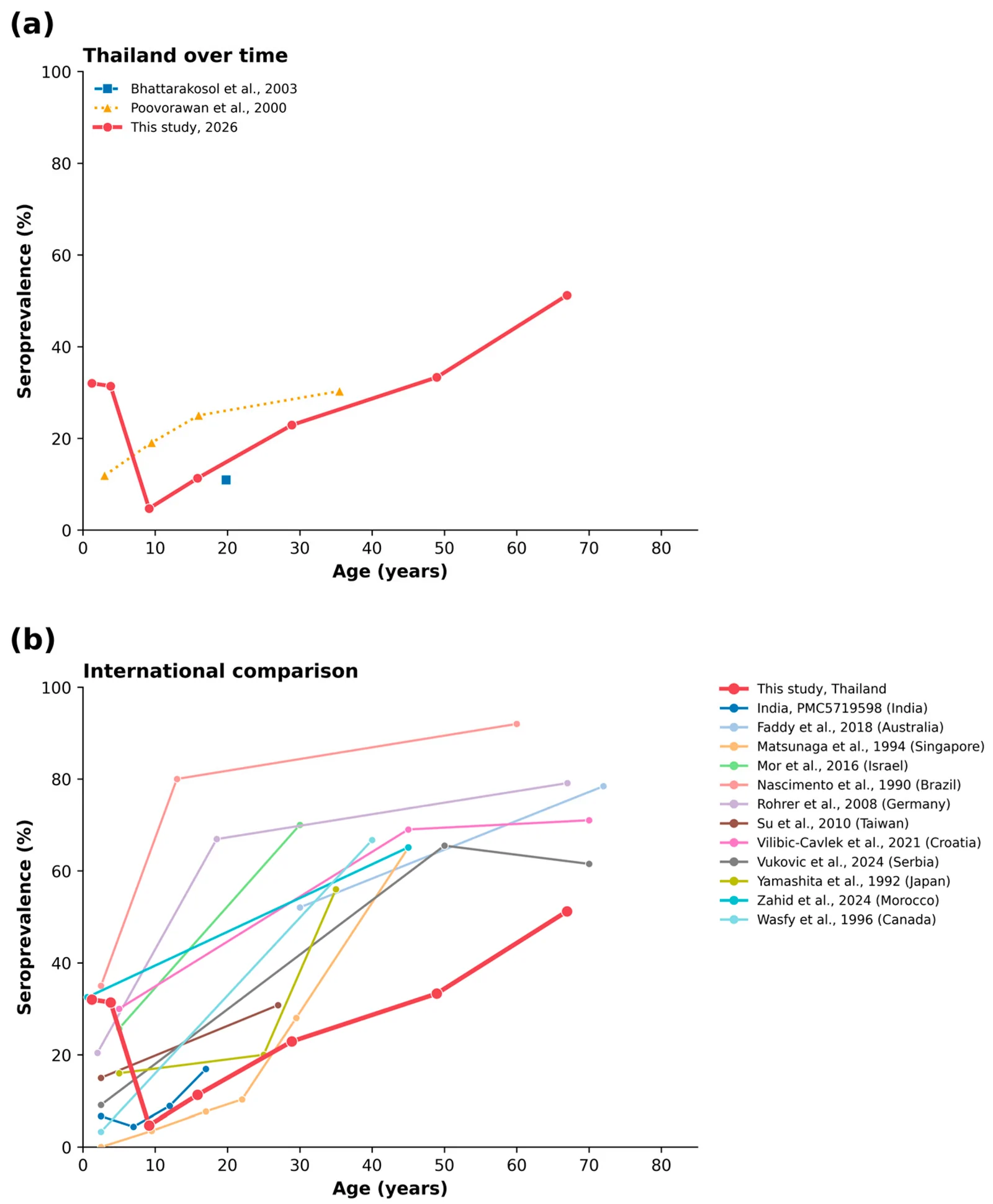
Age-specific parvovirus B19 seroprevalence in Thailand over time and in international comparison. (**a**) Age-specific seroprevalence of anti-parvovirus B19 IgG in the present study compared with the two previously published Thai serosurveys, Poovorawan et al. (2000) [28] (orange, triangles, dotted line) and Bhattarakosol et al. (2003) [30] (blue, squares, dashed line), spanning approximately two decades. The present study is shown in bold red; each line connects sequential age-group data points from the same source. (**b**) International comparison of age-specific parvovirus B19 IgG seroprevalence across 13 countries spanning five continents: Singapore [17], China [19], Japan [31], Israel [32], India [33] and Taiwan [34], (Asia); Germany [11], Croatia [12], and Serbia [21] (Europe); Australia [20] (Oceania); Brazil [35] and Canada [18] (the Americas); and Morocco [36] (Africa). The present study (Thailand) is highlighted in bold red for comparison. Each line connects the age-specific seroprevalence values reported in the corresponding source study. For the present study, each age group is plotted at the mean age of participants in that group. For comparator studies, closed age groups are plotted at their midpoints; open-ended age groups are plotted at the following ages: >35 years at 45 years (Singapore) and 50 years (Morocco), ≥51 years at 55 years (China; maximum donor age 59 years), >49 years at 65 years (Japan), ≥60 years at 70 years (Israel, Serbia, and Brazil), and ≥70 years at 75 years (Croatia). Characteristics of the comparator studies are provided in Supplementary Table S6.

Comparison with two previous Thai serosurveys showed a different pattern in the youngest age groups (Figure 4a). Poovorawan et al. reported seroprevalence of 11.9% among children aged 0 to 6 years and 19.1% among those aged 7 to 12 years in 2000 [28], whereas the corresponding estimates in the present study were 31.7% among children aged 0 to 5 years and 4.7% among those aged 6 to 12 years. Among young adults, Bhattarakosol et al. reported a seroprevalence of 10.9% among university students aged 18 to 24 years in 2003 [30], compared with 11.3% among participants aged 13 to 19 years and 22.9% among those aged 20 to 39 years in the present study.

In the descriptive international comparison, Thailand was generally positioned toward the lower end of the reported seroprevalence range from school age onward (Figure 4b; Supplementary Table S6). The Thai estimate of 4.7% among children aged 6 to 12 years was lower than most childhood estimates reported in the comparator studies, although the age strata were not directly equivalent across studies. In adulthood, the Thai estimate of 22.9% among participants aged 20 to 39 years was lower than estimates from Japan [31], Israel [32], Brazil [35], Germany [11], Croatia [12], Serbia [21], and Australia [20], broadly overlapped with estimates from Singapore [17], and was higher than estimates among Chinese blood donors aged 18 to 40 years [19]. Among participants aged ≥60 years, seroprevalence in Thailand was 51.2%, compared with 79.1% among German adults aged 65 to 69 years [11], 69.4% among Croatian adults aged ≥70 years [12], and 68.1% among Israeli adults aged ≥60 years [32].

## 4. Discussion

In this age-stratified cross-sectional serosurvey of 430 residual serum specimens from four provinces, one from each of Thailand’s four geographic regions, the overall seroprevalence of anti-parvovirus B19 IgG was 20.2%. To contextualise this estimate, we compared it with age-specific data from 13 countries spanning five continents (Figure 4b; Supplementary Table S6): Singapore [17], India [33], Taiwan [34], China [19], Japan [31], Israel [32], Germany [11], Croatia [12], Serbia [21], Australia [20], Brazil [35], Canada [18], and Morocco [36]. This overall estimate was generally lower than those reported in several population-based surveys from Europe and other temperate settings, where seroprevalence commonly increases during childhood and reaches substantially higher levels in adulthood [3,10,11,12,21], and was also lower than recent estimates from a tropical setting in South America [35], suggesting that the lower seroprevalence observed here is unlikely to be explained by climate alone. The present findings are broadly consistent with earlier Thai studies reporting relatively low B19V IgG seroprevalence among children and young adults [28,30]. Nevertheless, direct numerical comparisons across studies should be interpreted cautiously because of differences in age structure, sampling populations, calendar periods, assay platforms, and seropositivity cutoffs.

Several methodological and contextual factors may contribute to the comparatively low seroprevalence observed here relative to the temperate-country comparators. First, Thailand’s estimate did not stand in isolation: comparably low seroprevalence has also been reported from Singapore [17], India [33], Taiwan [34], and China [19], suggesting a broader regional pattern across East and Southeast Asia rather than a Thailand-specific anomaly, whereas the highest estimates were concentrated in the European, Australian, and South American comparators. Second, the Singapore estimate [17] was generated using an antigen-capture indirect immunosorbent assay developed before recombinant VP1/VP2-based ELISA platforms became standard, and may therefore have had lower analytical sensitivity than the assays used in more recent surveys; differences in seropositivity cutoffs and reporting conventions across studies compound this uncertainty. Third, sampling frames differed substantially across studies, spanning blood donors, pregnant women, hospital-based cohorts, and, in the present study, residual sera from a hepatitis serosurvey restricted to four non-metropolitan provinces; none of these frames is strictly population-representative, and the exclusion of Bangkok and other major urban centres, where population density and childcare or school contact patterns may favour more efficient B19V transmission, could further contribute to an underestimate relative to studies conducted in urban or national samples. Consequently, the lower seroprevalence observed in Thailand most likely reflects a combination of a genuine regional epidemiological pattern and cross-study methodological heterogeneity rather than a single, well-defined cause, and this distinction cannot be resolved with the present cross-sectional design.

The most notable finding was an apparent non-monotonic age-seroprevalence pattern. Seroprevalence was 31.7% among children aged 0–5 years, decreased to 4.7% among those aged 6–12 years, and subsequently increased across successive age groups, reaching 51.2% among participants aged 60 years and older. This pattern differs from the generally monotonic increase reported in many temperate populations, in which B19V seroprevalence rises rapidly during school age and subsequently plateaus in adulthood [10,11]. The low seroprevalence observed among Thai children aged 6–12 years may reflect differences in cohort-specific exposure, temporal variation in B19V circulation, or sampling variability. However, the cross-sectional design and absence of information on household composition, school attendance, childcare exposure, and contact with infected individuals prevent these possibilities from being distinguished.

The comparatively high seroprevalence among children aged 0 to 5 years warrants particularly cautious interpretation. Transplacentally acquired B19V IgG generally declines substantially during the first 6 to 12 months of life [11], so maternally derived antibody would be expected to produce seropositivity concentrated in infancy. However, when this age group was subdivided, seroprevalence was similar among children aged 1 to 2 and 3 to 5 years (33.3% and 31.4%, respectively), and only four participants were younger than 1 year (Supplementary Figure S2; Supplementary Table S3). Maternal antibody is therefore unlikely to account for most of the seropositivity observed in this age group. This interpretation is further supported by the low seroprevalence among adults aged 20 to 39 years (22.9%), which indicates that most mothers in this population would not have been able to transfer anti-B19V IgG to their infants. Several seropositive children aged 1 to 2 years had high IgG concentrations, compatible with true early-life infection, whereas most seropositive children aged 3 to 5 years had concentrations only slightly above the 5.5 IU/mL cut-off, in contrast to seropositive children aged 6 to 12 years, all of whom had concentrations of 10 IU/mL or higher. This distribution raises the possibility that low-level or non-specific reactivity near the assay cut-off contributed to the observed pattern. Other possible explanations include temporal variation in transmission, cohort effects, or sampling variation resulting from the modest number of children in this stratum. Because confirmatory testing using an alternative serological method was not performed, the relative contribution of these explanations cannot be determined from the present data. Prospective sampling combined with IgM, B19V DNA, or IgG avidity testing, together with confirmatory serological assays, would be required to distinguish recent infection, past infection, maternally acquired antibody, and assay-related reactivity.

From school age onward, seroprevalence increased progressively with advancing age, from 4.7% among participants aged 6–12 years to 11.3% at 13–19 years, 22.9% at 20–39 years, 33.3% at 40–59 years, and 51.2% at ≥60 years. This pattern is compatible with gradual accumulation of exposure over the life course rather than the early, rapidly saturating acquisition of immunity reported in many temperate populations. A similarly delayed accumulation of B19V immunity has been described in several tropical and subtropical Asian populations, including Malaysia and India [14,34]. Even among participants aged ≥60 years, however, approximately half remained non-seropositive, compared with approximately 68 to 79% reported among older adults in Germany, Croatia, and Israel [11,12,32]. These differences may reflect lower cumulative exposure, birth-cohort effects, temporal changes in transmission, or methodological differences between studies; the force of infection underlying this pattern is examined directly in the following section.

The estimated constant force of infection of 8.5 per 1000 person-years among participants aged 6 years and older should be interpreted as an average historical force of infection accumulated over the lifetimes of the participants, derived under the assumptions of lifelong immunity and time-constant transmission, rather than as a direct measure of B19V transmission intensity in Thailand in 2024. Moreover, because children younger than 6 years were excluded from model fitting, the estimate describes the age-seroprevalence relationship only among participants aged 6 years and older and does not account for the full age pattern observed in this study. With this caveat, the estimate was lower than the piecewise-constant estimates reported among Japanese blood donors and pregnant women, in which the highest forces of infection, 50 and 120 per 1000 person-years among those aged 0 to 4 and 5 to 9 years, respectively, were concentrated in early school age before declining to fewer than 10 per 1000 person-years in adulthood [31], and was likewise lower than the estimates described around age 7 to 9 years in the five European countries studied by Mossong et al. [10]. Neither a piecewise-constant nor a Farrington age-dependent model provided a statistically better fit than the constant model, and none of the 2000 bootstrap replicates placed the fitted peak before age 10 years. The present data therefore did not provide evidence for a concentrated school-age transmission peak of the kind reported in these settings, although the wide confidence interval for the estimated peak age and the limited size of several age strata preclude firm conclusions.

Because older participants accumulated their exposure over substantially different calendar periods, the observed age pattern may also reflect birth-cohort and historical-period effects, such as changes in family size, childcare and school contact patterns, and temporal variation in B19V circulation, which cannot be distinguished from age effects using cross-sectional data. Seroprevalence among older adults largely reflects infections acquired decades earlier; the constant estimate is therefore weighted toward historical exposure conditions, and contemporary transmission may be higher or lower than this average. For example, the low seroprevalence observed among children aged 6 to 12 years may indicate reduced exposure in recent birth cohorts, which a single time-constant parameter cannot capture. Differences in school-based contact patterns, population density, or other exposure determinants between Thailand and previously studied populations may also contribute to the observed age pattern.

The geometric mean concentration of anti-B19V IgG showed a broadly similar age-related pattern, decreasing from 3.10 IU/mL among children aged 0–5 years to 1.20 IU/mL among those aged 6–12 years and subsequently increasing to 6.42 IU/mL among participants aged ≥60 years. These estimates should be considered descriptive because GMCs were calculated for all participants, including non-seropositive individuals, and fixed values were assigned to concentrations below the lower limit of quantification and above the highest calibrator. In particular, the large number of results below 1 IU/mL and the upper censoring of concentrations above 100 IU/mL may have influenced the estimates. The population-level GMC therefore primarily reflects the proportion of seropositive individuals in each age group; among seropositive participants, IgG concentrations did not increase with age (Supplementary Table S7), and the present data do not provide evidence of antibody boosting.

The findings may have clinical relevance because a large proportion of adolescents and adults lacked detectable anti-B19V IgG, suggesting potential susceptibility to infection. Seroprevalence was only 22.9% among participants aged 20–39 years, indicating that more than three-quarters of participants in this age range lacked detectable anti-B19V IgG. Among women aged 20–39 years, 6 of 23 were seropositive; however, the small number of women in this stratum limits precise estimation of seroprevalence among women of reproductive age. Primary B19V infection during pregnancy can result in vertical transmission, fetal anemia, non-immune hydrops fetalis, and fetal loss, particularly when infection occurs during the first half of pregnancy [7,8]. These findings therefore support continued clinical awareness of B19V among pregnant women with compatible symptoms, unexplained fetal anemia, or non-immune hydrops fetalis. They should not, however, be interpreted as evidence supporting routine antenatal screening, which would require evaluation of clinical utility, feasibility, and cost-effectiveness. The substantial proportion of non-seropositive children and adults may also be relevant to individuals with chronic hemolytic disorders, including thalassemia, in whom B19V infection can precipitate transient aplastic crisis, and to immunocompromised individuals at risk of persistent infection and chronic anemia [3,5,6].

No statistically significant differences by sex or province were detected within age strata, consistent with several previous serosurveys [11,12,14]. However, these subgroup analyses were underpowered, particularly for province-specific comparisons. The absence of statistical significance should therefore not be interpreted as evidence of homogeneous B19V circulation across Thailand.

Several limitations should be considered. First, the cross-sectional, IgG-only design, without confirmatory testing by an alternative serological method, could not distinguish recent from remote infection or maternally acquired antibody, and the force-of-infection estimate relied on the assumptions of lifelong immunity and time-constant transmission, which cannot be verified without longitudinal or IgM, DNA, or avidity testing. Second, the specimens were residual samples from a hepatitis serosurvey covering one province per region and excluding major urban centers such as Bangkok; because specimens were allocated by age-specific sample size requirements rather than proportionally, were not compared with the parent population, and were not weighted, the overall seroprevalence of 20.2% reflects the study sample rather than a nationally representative estimate. Third, the study was not powered for subgroup analyses; several age- and province-specific subgroups were small, yielding wide confidence intervals and limited power to detect differences, and comparisons between age groups and provinces should therefore be interpreted with caution. Fourth, the international comparison relied on a non-systematic review of studies that differed in population, assay, sampling design, and study period; differences from other countries may therefore partly reflect methodology rather than lower transmission in Thailand. Fifth, B19V is not notifiable in Thailand, so the epidemic phase during the 2024 sampling period could not be determined; short-term variation in transmission may therefore have influenced the estimates, particularly in the youngest age groups.

In conclusion, this serosurvey identified low anti-B19V IgG seroprevalence from school age onward among participants sampled from four provinces in Thailand, with a large proportion of adults lacking detectable anti-B19V IgG. The unexpectedly high seroprevalence among young children produced a non-monotonic age pattern that could not be fully explained by the available cross-sectional data. Larger population-based studies using standardized serological thresholds and complementary IgM, molecular, or avidity testing are needed to confirm the age pattern and clarify B19V transmission in Thailand.

## Figures and Tables

**Table 1:** Demographic characteristics of study participants by age group.

Age Group (Years)	Age, Mean ± SD	Sex		Total (n)
		Male (n)	Female (n)	
0–5	2.75 ± 1.48	33	27	60
6–12	9.15 ± 1.82	47	60	107
13–19	15.86 ± 1.99	60	64	124
20–39	28.90 ± 5.82	25	23	48
40–59	48.94 ± 5.96	16	32	48
≥60	66.95 ± 4.71	16	27	43
Total	22.62 ± 20.11	197	233	430

Note: Participants were 6 months to 80 years of age; ages under 1 year are recorded as 0.

**Table 2:** Analysis of demographic factors and parvovirus B19 seroprevalence by age group.

Age Range (Years)	Parvovirus B19 Serostatus	Sex			Province				
		Male	Female	*p* Value	Ayutthaya	Trang	Buri Ram	Uttaradit	*p* Value
0–5	+	11	8		3	3	7	6	
	−	22	19	0.759	12	12	9	8	0.294 ‡
6–12	+	1	4		2	2	1	0	
	−	46	56	0.382 †	25	25	25	27	0.660 ‡
13–19	+	10	4		4	6	2	2	
	−	50	60	0.067	27	25	29	29	0.366 ‡
20–39	+	5	6		1	4	4	2	
	−	20	17	0.616	11	8	8	10	0.471 ‡
40–59	+	3	13		2	7	4	3	
	−	13	19	0.130	10	5	8	9	0.220 ‡
≥60	+	8	14		5	7	6	4	
	−	8	13	0.808	6	4	5	6	0.797 ‡

Note: Data are presented as the number of participants in each category. + = seropositive (≥5.5 IU/mL); − = non-seropositive (<5.5 IU/mL, including borderline results of 4 to <5.5 IU/mL, *n* = 16). *p* values were obtained by the χ^2^ test; † Fisher’s exact test and ‡ Monte Carlo χ^2^ test (20,000 replicates) were used where any expected cell count was <5. indicates that no test could be computed because one category was empty. *p* < 0.05 was considered statistically significant. One province was included from each of the four regions of Thailand: Ayutthaya (central), Trang (southern), Buri Ram (northeastern), and Uttaradit (northern).

## Data Availability

The de-identified data supporting the findings of this study are available from the corresponding author on request. The analysis code used for the force-of-infection models is also available on request.

## Supplementary Materials

The following supporting information can be downloaded at https://www.mdpi.com/article/10.3390/tropicalmed11100291/s1, Figure S1: Force-of-infection model comparison. (a) Observed proportion seropositive by age (points, 95% CI) with three fitted models: constant, piecewise-constant, and Farrington. Open circles (grey shading) mark the 0–2 and 3–5 year groups excluded from fitting. (b) Estimated force of infection by age under each model. The constant model gave 8.5 per 1000 person-years; neither alternative model improved the fit, and none of the 2000 bootstrap replicates placed the peak before age 10 years. Figure S2: Anti-parvovirus B19 IgG among children aged 0 to 12 years. (A) Distribution of IgG concentrations by age group (<1, 1 to 2, 3 to 5, and 6 to 12 years). Seroprevalence with Clopper-Pearson exact 95% confidence intervals is shown above each bar. (B) Individual IgG concentrations by single year of age on a log10 scale; each point represents one participant, colored by IgG category as in panel A. Horizontal black dashes indicate the median for each year of age. The dashed and dotted horizontal lines mark 5.5 and 4.0 IU/mL, respectively, and the shaded area indicates children aged 0 to 5 years. Values <1 IU/mL were assigned 0.5 IU/mL and values >100 IU/mL were assigned 100 IU/mL; Table S1: Pairwise comparisons of anti-parvovirus B19 IgG concentration by age group; Table S2: Comparison of serocatalytic models fitted to participants aged 6 years and older (*n* = 370); Table S3: Observed versus model-predicted parvovirus B19 seroprevalence by age group under the constant force-of-infection model fitted to participants aged 6 years and older; predicted values for children aged 0 to 5 years represent model extrapolation; Table S4: Anti-parvovirus B19 IgG serostatus and concentrations among children aged 0 to 12 years, by narrower age group. Data are presented as number of participants unless otherwise indicated; IgG categories are given in IU/mL; Table S5: Sensitivity analysis of parvovirus B19 seroprevalence and force of infection according to the classification of borderline results; Table S6: Characteristics of the comparator studies included in the descriptive international comparison (Figure 4b); Table S7: Geometric mean concentration (GMC) of anti-parvovirus B19 IgG among seropositive participants, by age group.
