# Supplementary material for: Age-Specific Seroprevalence of Human Parvovirus B19 in Thailand: A Cross-Sectional Study Across Four Geographic Regions with Global Comparison
Source: Trop Med Infect Dis. 2026 Oct 13;11(10):291. doi: 10.3390/tropicalmed11100291 (PMC13709610; doi:10.3390/tropicalmed11100291)
Supplement: Supplementary file 1 [file tropicalmed-11-00291-s001.zip › tropicalmed-4556718-supplementary.pdf]

Age-Specific Seroprevalence of Human Parvovirus B19 in Thailand: A Cross-Sectional Study Across Four Geographic Regions with Global Comparison

Pornjarim Nilyanimit [1], Jiratchaya Puenpa [1], Nungruthai Suntronwong [1], Sirapa Klinfueng [1], Ritthideach Yorsaeng [1], Thananrat Thongmee [1] and Yong Poovorawan [1,2,*]

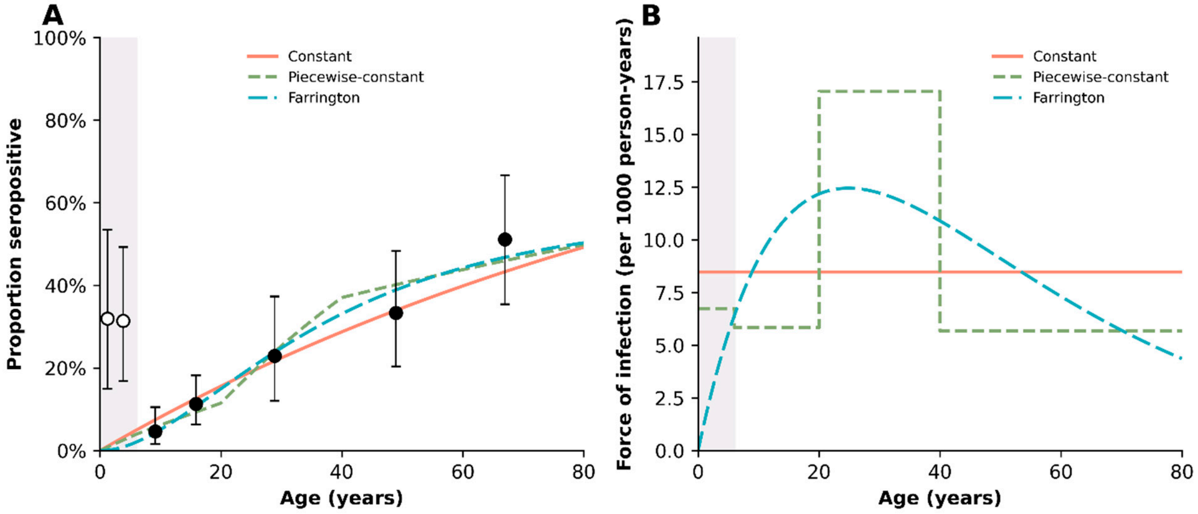

Supplementary Figure S1. Force-of-infection model comparison. (a) Observed proportion seropositive by age (points, 95% CI) with three fitted models: constant, piecewise-constant, and Farrington. Open circles (grey shading) mark the 0–2 and 3–5 year groups excluded from fitting. (b) Estimated force of infection by age under each model. The constant model gave 8.5 per 1,000 person-years; neither alterna-tive model improved the fit, and none of the 2,000 bootstrap replicates placed the peak before age 10 years.

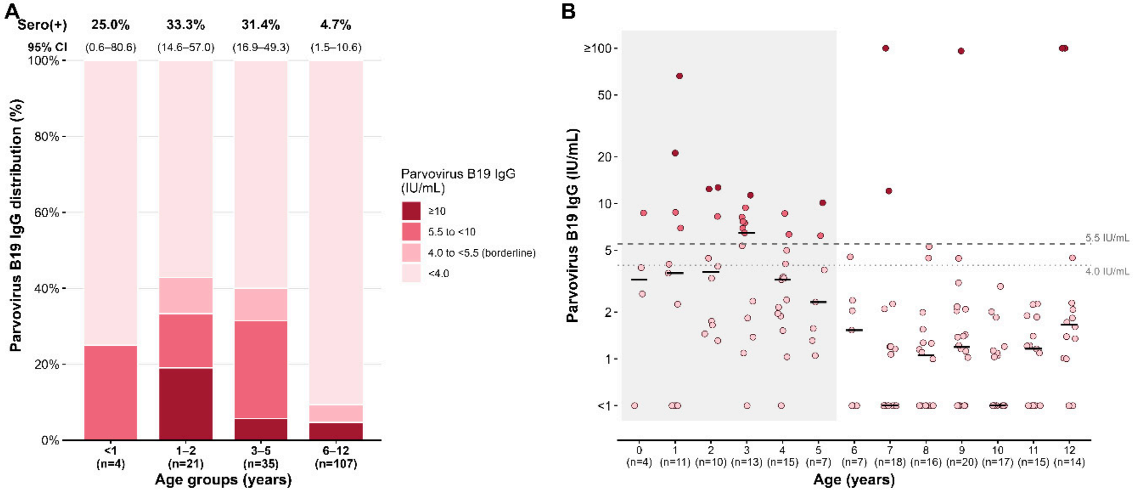

Supplementary Figure S2. Anti-parvovirus B19 IgG among children aged 0 to 12 years. (A) Distribution of IgG concentrations by age group (<1, 1 to 2, 3 to 5, and 6 to 12 years). Seroprevalence with Clopper-Pearson exact 95% confidence intervals is shown above each bar. (B) Individual IgG concentrations by single year of age on a log10 scale; each point represents one participant, colored by IgG category as in panel A. Horizontal black dashes indicate the median for each year of age. The dashed and dotted horizontal lines mark 5.5 and 4.0 IU/mL, respectively, and the shaded area indicates children aged 0 to 5 years. Values <1 IU/mL were assigned 0.5 IU/mL and values >100 IU/mL were assigned 100 IU/mL.

Supplementary Table S1. Pairwise comparisons of anti-parvovirus B19 IgG concentration by age group.

Age group comparison	Adjusted *p* value	Significance
0-5 vs 6-12	<0.0001	****
0-5 vs 13-19	<0.0001	****
0-5 vs 20-39	0.1033	ns
0-5 vs 40-59	1.0000	ns
0-5 vs ≥60	1.0000	ns
6-12 vs 13-19	1.0000	ns
6-12 vs 20-39	0.9765	ns
6-12 vs 40-59	0.0027	**
6-12 vs ≥60	<0.0001	****
13-19 vs 20-39	0.3375	ns
13-19 vs 40-59	0.0004	***
13-19 vs ≥60	<0.0001	****
20-39 vs 40-59	1.0000	ns
20-39 vs ≥60	0.0370	*
40-59 vs ≥60	1.0000	ns

*Pairwise differences in log10-transformed anti-B19V IgG concentration distributions across the six age groups were assessed using the Kruskal-Wallis test ($H = 68.53$, $df = 5$, $p < 0.0001$) followed by Dunn's post-hoc test with Bonferroni adjustment for multiple comparisons (15 pairwise comparisons). ns, not significant; $p < 0.05$; **$p < 0.01$; ***$p < 0.001$; ****$p < 0.0001$.*

Supplementary Table S2. Comparison of serocatalytic models fitted to participants aged 6 years and older (n = 370).

Model	Parameters (k)	Log-likelihood	AIC	ΔAIC	BIC	ΔBIC	LRT vs constant, p
Constant FOI	1	−150.63	303.26	0.16	307.17	0.00	Reference
Piecewise-constant (0 to <6, 6 to <20, 20 to <40, ≥40 years)	4	−148.93	305.86	2.76	321.52	14.35	0.335
Farrington (γ = 0)	2	−149.55	303.10	0.00	310.93	3.76	Not applicable

All models were fitted to the same analytic dataset of participants aged 6 years and older (n = 370). In each model, the FOI was assumed to operate from birth; the 0 to <6 year band of the piecewise-constant model therefore represents exposure accumulated before age 6 years by participants in the analytic dataset and was estimated indirectly, without data from children younger than 6 years. The constant model is nested within the piecewise-constant model (all four band-specific rates constrained to be equal), which was compared with it using a LRT with 3 degrees of freedom (four versus one parameter). The Farrington model (with γ fixed at 0) is not nested within the constant model and was therefore compared using AIC and BIC only; differences of less than 2 units were considered to indicate no meaningful difference in fit. ΔAIC and ΔBIC are differences from the lowest value among the three models. AIC, Akaike information criterion; BIC, Bayesian information criterion; FOI, force of infection; LRT, likelihood ratio test.

Supplementary Table S3. Observed versus model-predicted parvovirus B19 seroprevalence by age group under the constant force-of-infection model fitted to participants aged 6 years and older; predicted values for children aged 0 to 5 years represent model extrapolation.

Age group (years)	n	Seropositive (k)	Mean age (years)	Observed seroprevalence, % (95% CI)	Predicted seroprevalence, %	Observed/predicted ratio	Included in primary FOI fit
0-2	25	8	1.24	32.0 (14.9–53.5)	1.1	30.60×	No
3-5	35	11	3.83	31.4 (16.9–49.3)	3.2	9.84×	No
6-12	107	5	9.15	4.7 (1.5–10.6)	7.5	0.63×	Yes
13-19	124	14	15.86	11.3 (6.3–18.2)	12.6	0.90×	Yes
20-39	48	11	28.90	22.9 (12.0–37.3)	21.7	1.05×	Yes
40-59	48	16	48.94	33.3 (20.4–48.4)	34.0	0.98×	Yes
≥60	43	22	66.95	51.2 (35.5–66.7)	43.3	1.18×	Yes

Predicted seroprevalence was calculated from the constant force-of-infection model (λ = 8.5 per 1,000 person-years; 95% CI, 6.7 to 10.8) fitted to participants aged 6 years and older. Observed seroprevalence is shown with Clopper-Pearson exact 95% confidence intervals. The 0 to 2 and 3 to 5 year age groups were excluded from the model fit because seroprevalence declined between the 3 to 5 and 6 to 12 year groups, which is incompatible with the assumption of lifelong immunity; predicted values for these groups were therefore obtained by extrapolating the fitted model below age 6 years. The corresponding ratios of observed to predicted seroprevalence (30.6× and 9.84×, respectively) reflect the discrepancy between the observed data and this extrapolation and should not be interpreted as a measure of transmission intensity in these age groups. FOI, force of infection; CI, confidence interval.

Supplementary Table S4. Anti-parvovirus B19 IgG serostatus and concentrations among children aged 0 to 12 years, by narrower age group. Data are presented as number of participants unless otherwise indicated; IgG categories are given in IU/mL.

Age group	*n*	Negative (<4.0)	Borderline (4.0 to <5.5)	Positive (5.5 to <10)	Positive (≥10)	Seroprevalence, % (95% CI)[a]	Median IgG, IU/mL (IQR)[b]
<1 year	4	3	0	1	0	25.0 (0.6 to 80.6)	3.24 (2.09 to 5.07)
1 to 2 years	21	12	2	3	4	33.3 (14.6 to 57.0)	3.56 (1.45 to 8.26)
3 to 5 years	35	21	3	9	2	31.4 (16.9 to 49.3)	3.30 (1.70 to 6.40)
6 to 12 years	107	97	5	0	5	4.7 (1.5 to 10.6)	1.10 (0.50 to 1.86)

Serostatus was classified according to the manufacturer's instructions: negative, <4.0 IU/mL; borderline, 4.0 to <5.5 IU/mL; and positive, ≥5.5 IU/mL. The ≥10 IU/mL stratum is a descriptive category only and does not correspond to a validated diagnostic threshold. Children aged 6 to 12 years are shown for reference. Seroprevalence did not differ between children aged 1 to 2 and 3 to 5 years (Fisher's exact test, P = 1.00), and IgG concentration distributions did not differ among the three groups aged <1, 1 to 2, and 3 to 5 years (Kruskal-Wallis test, p = 0.97). Compared with children aged 6 to 12 years, children aged 0 to 5 years had higher seroprevalence (31.7% vs 4.7%; Fisher's exact test, P < 0.001) and higher IgG concentrations (Mann-Whitney U test, p < 0.001). CI, confidence interval; IQR, interquartile range.

[a] *95% CIs were calculated using the Clopper-Pearson exact method.*

[b] *Values <1 IU/mL were assigned 0.5 IU/mL, and values >100 IU/mL were assigned 100 IU/mL.*

Supplementary Table S5. Sensitivity analysis of parvovirus B19 seroprevalence and force of infection according to the classification of borderline results.

	Primary analysis[a]	Borderline reclassified as seropositive	Borderline excluded
Number of specimens analysed	430	430	414
Number seropositive	87	103	87
**Seroprevalence, % (95% CI)**			
Overall	20.2 (16.5 to 24.3)	24.0 (20.0 to 28.3)	21.0 (17.2 to 25.3)
0 to 5 years	31.7 (20.3 to 45.0)	40.0 (27.6 to 53.5)	34.5 (22.2 to 48.6)
6 to 12 years	4.7 (1.5 to 10.6)	9.3 (4.6 to 16.5)	4.9 (1.6 to 11.1)
13 to 19 years	11.3 (6.3 to 18.2)	12.1 (6.9 to 19.2)	11.4 (6.4 to 18.4)
20 to 39 years	22.9 (12.0 to 37.3)	22.9 (12.0 to 37.3)	22.9 (12.0 to 37.3)
40 to 59 years	33.3 (20.4 to 48.4)	37.5 (24.0 to 52.6)	34.8 (21.4 to 50.2)
≥60 years	51.2 (35.5 to 66.7)	58.1 (42.1 to 73.0)	55.0 (38.5 to 70.7)
**Constant force of infection**			
Participants aged ≥6 years, n	370	370	359
Per 1,000 person-years (95% CI)	8.5 (6.7 to 10.8)	10.1 (8.1 to 12.6)	8.9 (7.0 to 11.3)

Sixteen specimens had borderline IgG concentrations (4.0 to <5.5 IU/mL): five among participants aged 0 to 5 years, five aged 6 to 12 years, one aged 13 to 19 years, two aged 40 to 59 years, and three aged 60 years and older. Seroprevalence 95% CIs were calculated using the Clopper-Pearson exact method. The constant force of infection was estimated using a serocatalytic model (binomial generalised linear model with a complementary log-log link and log(age) offset) fitted to participants aged 6 years and older; 95% CIs are Wald intervals on the log scale. CI, confidence interval.

[a] Borderline results classified as non-seropositive.

Supplementary Table S6. Characteristics of the comparator studies included in the descriptive international comparison (Figure 4B).

Country	Study [ref]	Study period	Study population	Sample size (n)	Age groups reported (years)	Assay	Seropositivity threshold
Singapore	Matsunaga et al., 1994 [17]	February to July 1993	Healthy volunteers from the 1993 serosurvey on vaccine-preventable diseases (non-random)[a]	600	0 to 4, 5 to 14, 15 to 19, 20 to 24, 25 to 29, 30 to 34, 35 to 39, 40 to 49, ≥50	Antigen-capture indirect ELISA (anti-B19 monoclonal antibody, native B19 antigen)	NR
India	Viswanathan et al., 2017 [33]	2009	Archived sera, Pune district (hospital staff, general practitioners, school children and staff)	668	<5, 5 to 9, 10 to 14, 15 to 19, 20 to 60	SERION ELISA classic (Virion\Serion) and NovaLisa (NovaTec)	NR
Taiwan	Su et al., 2010 [34]	2004	Stored sera from healthy volunteers aged 1 to 29, Taipei, randomly selected by age group	277	1 to 4, 5 to 9, 10 to 14, 15 to 19, 20 to 24, 25 to 29	Biotrin Parvovirus B19 IgG EIA (recombinant VP2)	Manufacturer's cut-off
China	Lu et al., 2024 [19]	July 2022 to April 2023	Blood donors, Suzhou Blood Center	893	18 to 30, 31 to 40, 41 to 50, ≥51	Virion\Serion ELISA classic	>5 IU/mL (3 to 5 IU/mL equivocal, retested)
Japan	Nabae et al., 2014 [31]	2004 to 2007	Healthy individuals randomly sampled in the National Epidemiological Surveillance of Vaccine-Preventable Diseases, Fukuoka and Saga prefectures	1,000	0 to 4, 5 to 9, 10 to 14, 15 to 19, 20 to 29, 30 to 39, 40 to 49, >49[b]	Denka Seiken IgG EIA	Antibody index ≥1.00
Israel	Mor et al., 2016 [32]	January 2009 to December 2010	Residual sera from the National Sera Bank (diagnostic laboratories and blood donors), age-stratified random sample	1,008	0 to 9, 10 to 19, 20 to 29, 30 to 39, 40 to 49, 50 to 59, ≥60	DiaSorin Biotrin Parvovirus B19 IgG EIA	Manufacturer's cut-off; equivocal classified as negative
Germany	Röhrer et al., 2008 [11]	Adults 1997 to 1998; children 1999 to 2006	Adults from the German National Health Examination Survey (18 to 79); healthy children and adolescents recruited by paediatricians, Thuringia (0 to 18)	6,583 adults; 649 children	Yearly in children; 5-year groups in adults	recomWell Parvovirus B19 IgG ELISA (VP1/VP2; Mikrogen)	Manufacturer's cut-off controls; borderline classified as negative
Croatia	Vilibić-Čavlek et al., 2021 [12]	2010 to 2021	Consecutive sera tested at the Croatian Institute of Public Health (children and adolescents, general adults, hemodialysis patients, liver transplant recipients)[c]	1,538	<0.5, 0.5 to 9, 10 to 19, 20 to 29, 30 to 39, 40 to 49, 50 to 59, 60 to 69, ≥70	Euroimmun Anti-Parvovirus B19 ELISA (IgG)	>5.5 RU/mL (4 to 5.5 RU/mL borderline)
Serbia	Vuković et al., 2024 [21]	September 2008 to September 2023	Outpatient and inpatient sera referred for PVB19 testing, Institute of Public Health of Vojvodina[c]	7,325	<1, 1 to 4, 5 to 9, 10 to 19, 20 to 39, 40 to 59, ≥60	Euroimmun and Virion\Serion ELISA	Euroimmun ratio >1.1; Virion\Serion >5 IU/mL; equivocal excluded

Country	Study [ref]	Study period	Study population	Sample size (n)	Age groups reported (years)	Assay	Seropositivity threshold
Australia	Faddy et al., 2018 [20]	Donors June to September 2016; children March 2015 to June 2016	Blood donors stratified by age, sex, and state; paediatric patients presenting for non-infectious investigations, Queensland	2,221 donors; 223 children	0 to 4, 5 to 9, 10 to 15, 16 to 24, 25 to 34, 35 to 44, 45 to 54, 55 to 64, 65 to 80	Euroimmun Anti-Parvovirus B19 ELISA (IgG)	Ratio ≥1.1; borderline (0.8 to <1.1) classified as positive[d]
Brazil	Soares et al., 2026 [35]	May 2021 to May 2023	Community-based, age-stratified, non-probabilistic sample of residents recruited at schools, universities, and community sites, Manaus	450	7 to 19, 20 to 29, 30 to 39, 40 to 49, 50 to 59, ≥60	Euroimmun ELISA Classic Parvovirus B19 (IgG)	>5.5 IU/mL
Canada	Wasfy et al., 1996 [18]	NR (published 1996)	Paediatric in- and outpatients treated for non-rash illnesses, Hospital for Sick Children, Toronto, and adult Red Cross blood donors, Ontario; randomly selected by sex	210	1 to 5, 5 to 15, 15 to 25, 25 to 35, 35 to 45, 45 to 55, 55 to 65	In-house indirect IgG EIA (baculovirus-expressed recombinant B19 antigen)	Absorbance >0.025 and positive/negative ratio >2.00; indeterminate classified as negative
Morocco	Zahid et al., 2024 [36]	March 2019 to July 2022	Patients referred for PVB19 serology, Central Virology Laboratory, Ibn Sina University Hospital, Rabat (infants <6 months excluded)[c]	343	0.6 to 5, 6 to 16, 17 to 35, >35	Chorus anti-VP2 IgG ELISA (Diesse)	Index >1.2 (0.8 to 1.2 equivocal, excluded)

One study was selected per country. Age-group midpoints were used for plotting because individual-level ages were not available for comparator studies. Studies differed in population, sampling design, assay, seropositivity threshold, and study period, so direct numerical comparisons should be interpreted with caution. ELISA, enzyme-linked immunosorbent assay; EIA, enzyme immunoassay; NR, not reported in the source publication.

[a] Volunteers responding to a public announcement; the authors noted that the sample was not random.

[b] Age-specific seroprevalence was reported graphically only; values were digitised from Figure 2A of the source publication (the resulting mean across age groups, 54.7%, agrees with the reported overall seroprevalence of 54.3%).

[c] Samples were tested for clinical indications rather than drawn from a population-based sampling frame.

[d] Borderline results were classified as seropositive in this study, whereas borderline results were classified as seronegative in the present study and in several other comparator studies.

Supplementary Table S7. Geometric mean concentration (GMC) of anti-parvovirus B19 IgG among seropositive participants, by age group.

Age group (years)	Tested, *n*	Seropositive, *n*	GMC among seropositive, IU/mL (95% CI)[a]	Median (IU/mL)	Values >100 IU/mL, *n*[b]
0 to 5	60	19	9.84 (7.55 to 12.83)	8.64	0
6 to 12	107	5	64.95 (20.17 to 209.15)	100.00	3
13 to 19	124	14	43.70 (26.93 to 70.90)	70.60	2
20 to 39	48	11	36.57 (18.46 to 72.45)	23.98	5
40 to 59	48	16	40.52 (24.06 to 68.26)	72.24	4
≥60	43	22	29.24 (19.84 to 43.08)	23.22	2

Note: Seropositivity was defined as an IgG concentration ≥5.5 IU/mL. Among seropositive participants aged 6 years and older, IgG concentrations did not differ across age groups (Kruskal-Wallis test, $p = 0.27$). CI, confidence interval; GMC, geometric mean concentration.

[a] Calculated from log10-transformed concentrations; 95% CIs were derived on the log scale using the t distribution and back-transformed.

[b] Values above the highest calibrator (>100 IU/mL) were assigned 100 IU/mL; GMCs in age groups with such values are therefore conservative (underestimated), and the estimate for the 6 to 12 year group is based on only five participants.
